# Repurposing Success: Real-World Validation of a Novel Cephalosporin Decontamination Protocol Using Chlorine Dioxide and Disc Diffusion Testing

**DOI:** 10.1177/15356760251388996

**Published:** 2025-11-13

**Authors:** Rhys Tancock-Jones, Sofianos Kyriakidis, Brett Cole, Jeremy Martens

**Affiliations:** 1 Royal Society of Biology, London, United Kingdom.; 2 Remedica Ltd, Limassol, Cyprus.; 3 Association for Biosafety for Australia and New Zealand (ABSANZ), Victoria, Australia.; 4 AMAL Analytical (Australia) Pty Ltd, Victoria, Australia.

**Keywords:** cephalosporin decontamination, chlorine dioxide gas, decontamination, beta-lactams, microbial challenge

## Abstract

**Introduction:**

Beta-lactam manufacturing facilities face strict regulatory challenges to repurposing for non-beta-lactam production due to the high risk of cross contamination. This study introduces a novel decontamination protocol for cephalosporin facilities, combining chlorine dioxide (ClO_2_) treatment with disc diffusion testing to verify the complete degradation and inactivation of beta-lactam residues in a real-world setting.

**Methods:**

Antimicrobial susceptibility discs (ASDs) contaminated with cephalosporins (Cefalexin, Cefaclor, Cefixime, Cefuroxime) were exposed to both aqueous (400 ppm for 24 h) and gaseous ClO_2_ (5 mg/L for >5 h) in separate cycles, achieving a minimum cumulative exposure of 9600 ppm-hours in both. Post-treatment, replicates (4 = aqueous cycle, 5 = gaseous cycle) of each cephalosporin were analyzed using liquid chromatography–tandem mass spectrometry (LC-MS/MS) and microbial challenge testing to assess degradation and biological inactivation, respectively.

**Result:**

For all, LC-MS/MS confirmed cephalosporin degradation below the reporting limit (<0.02 μg/disc), while microbial challenge testing demonstrated no significant biological activity with <1 mm bacterial inhibition. Microbial challenge and LC-MS/MS results showed significant agreement (Cohen’s κ = 1.00) in both aqueous and gaseous ClO_2_ cycles.

**Verification:**

Gaseous ClO_2_ application at 9600 ppm-hours was applied to a 450 m^2^ cephalosporin facility. Analysis confirmed that cephalosporin residues were degraded to levels below the acceptable limits (<1 μg/disc) with microbial challenge testing supporting the success of the cycle, with no significant biological activity remaining in the discs placed throughout the facility.

**Conclusion:**

This study presents the first successful protocol for cephalosporin facility decontamination using gaseous ClO_2_ in a real-world setting.

## Introduction

Cephalosporins are a widely used class of beta-lactam antibiotics, critical in treating various bacterial infections due to their broad-spectrum activity.^1–5^ They account for a significant proportion of antibiotics produced globally, and their use spans across various health care sectors. In the pharmaceutical industry setting, the production of beta-lactam drugs is required to be conducted in dedicated and self-contained facilities.^1–4,6^ The underlying rational of this fundamental control is to drastically reduce the risk of cross contamination to non-beta-lactam drugs, as cephalosporins and other beta-lactam antibiotics are known to trigger hypersensitivity reactions in sensitive individuals, which in some cases can be life threatening through anaphylaxis.^1,3–6^

Pharmaceutical manufacturers face substantial challenges when attempting to repurpose dedicated facilities and equipment previously used for beta-lactam production. Failure to adequately decontaminate them poses an unacceptable health risk to patients and can lead to severe regulatory enforcement including product recalls and potential suspension of manufacturing licenses.^5^ In the absence of a consensus for deriving health-based exposure limits for beta-lactams in the pharmaceutical industry setting, manufacturers who wish to repurpose their dedicated facility and equipment are required to prove the absence of detectable residues against the most sensitive analytical methods available.^6–9^ This is often problematic for two main reasons. First, it is highly dependent on the analytical method and technology available. Second, it does not adequately address the critical biological endpoint, which ultimately concerns patient safety, meaning it does not address the research question, whether any undetectable residues left remain pharmacologically active to elicit an immune-mediated reaction in sensitive patients.

Traditional liquid cleaning methods have limitations, particularly regarding their inability to reach all surfaces within a facility, especially in complex layouts and confined spaces.^10–13^

Manual deep cleaning often fails to ensure complete decontamination, which increases the risk of residual cephalosporins persisting in hard-to-reach areas. Takahashi et al. (2008) reported that, for the adequate control of this risk, the hard-to-reach equipment and installations had to be removed from a cephalosporin synthesis facility prior to its decontamination.^12^ Therefore, a robust decontamination method that reaches all parts of the facility, including equipment and ventilation systems, is necessary to ensure the complete removal of beta-lactam residues.

Chlorine dioxide (ClO_2_) has emerged as a promising solution for decontaminating pharmaceutical facilities.^4^ Its application as a dry gas (dClO_2_) is particularly advantageous as it can permeate complex environments, ensuring complete surface contact and efficient degradation of beta-lactam compounds. Previous studies, such as those conducted by Lorcheim et al. (2009), demonstrated the effectiveness of dClO_2_ in degrading various beta-lactam residues, including ampicillin and penicillin G.^5,9,14^ However, while effective in controlled environments, these studies did not fully address the application of ClO_2_ in real-world pharmaceutical settings, where facility complexity and contamination levels vary significantly.

A more recent study by Tancock-Jones et al. (2024) expanded on this work, demonstrating that dClO_2_ could successfully degrade cephalosporins in both gaseous and aqueous forms under controlled laboratory conditions.^5^ The research confirmed that exposure to 9600 ppm-hours of dClO_2_ effectively degraded cephalosporin residues to levels below detectable limits.^5^ Despite these promising results, further research is necessary to validate the scalability and practical application of this decontamination method in real-world pharmaceutical facilities.

Building on the work of Tancock-Jones et al., this study seeks to verify the effectiveness of dClO_2_ decontamination in a large-scale pharmaceutical facility, focusing on cephalosporin residues. A key innovation in this research is the use of antimicrobial susceptibility discs (ASDs) as dual-function chemical and biological indicators. The ASDs allow for simultaneous verification of both chemical degradation of cephalosporin compounds and biological inactivation of any remaining bioactive materials. This approach not only streamlines the decontamination process but also offers a more comprehensive validation method, ensuring both chemical degradation and biological inactivation of contaminants.

The overarching goal of this study is to establish a novel cleaning validation protocol that integrates dClO_2_ decontamination with dual verification methods. This protocol is designed to be scalable and adaptable to a wide range of pharmaceutical environments, addressing the need for effective, reliable decontamination methods for facilities transitioning from beta-lactam production to other manufacturing processes. By validating this approach in a real-world facility, this research aims to provide a practical, industry-wide, and regulatory acceptable solution for cephalosporin decontamination.

## Methods

The study was conducted in two phases: validation and verification. In the validation phase, both aqueous and gaseous ClO_2_ were used to degrade and inactivate each cephalosporin compound (Table 1) in laboratory-controlled conditions using 9600 ppm-hours exposure. In the verification phase, results were retrieved from a real-world facility decontamination event using dClO_2_ at 9600 ppm-hours. ASDs were employed to assess both chemical degradation and biological inactivation in both validation and verification phases.

**Table 1.** Details of cephalosporin compounds used

Compound	Molecular weight	Formula	Generation	Source	Starting amount^a^
Cefalexin	347.4	C_16_H_17_N_3_O_4_S	One	Bio-Rad	30 μg
Cefaclor	367.8	C_15_H_14_CIN_3_O_4_S	Two	OxoID	34 μg
Cefuroxime	424.4	C_16_H_16_N_4_NaO_8_S	Two	Bio-Rad	30 μg
Cefixime	453.5	C_16_H_15_N_5_O_7_S_2_	Three	Bio-Rad	5 μg

a Certified during release—subject to variability.

### Validation Phase

#### Detection limits

An ultra-performance liquid chromatography–tandem mass spectrometry (UPLC-MS/MS) recovery method was developed for each of the cephalosporins directly from pre-impregnated ASDs being studied. The analytical method was developed on a Waters Acquity I-Class UPLC system linked to a Waters TQs micro mass spectrometer; separation was performed on an Acquity BEH C18 column (1.7 μm, 2.1 × 50 mm) using H_2_O (0.1% formic acid)/acetonitrile elution. Analytes were quantified through multiple reaction monitoring in positive ion mode with the following reactions (Table 2).

**Table 2.** Analyte quantification

Compound	Quantifier	Qualifier 1	Qualifier 2
Cefalexin	348	106	158
Cefaclor	368	106	118
Cefuroxime	442	336	364
Cefixime	454	126	284

Quantifier, quantifier ion mass-to-charge ratio (m/z) of the target compound; Qualifier, qualifier ion mass-to-charge ratio (m/z) of the secondary ion after reaction, used to confirm the presence of analyte—appears in a set ratio with the quantifier ion.

#### Sample preparation

For each cephalosporin compound, ASDs were placed in a 50 mL polypropylene vial with 20 mL extraction solution (0.1% formic acid/acetonitrile) added. The vial was capped and placed on a rotational mixer for 60 min. The vial was removed from the mixer, then a portion of solution was filtered through a 0.22 μm filter into an autosampler vial ready for analysis.

#### Phase treatment disc preparation

Commercially available ASDs were used for the four compounds Cefalexin, Cefaclor, Cefixime and Cefuroxime. Each of the ASDs were pre-impregnated with known concentrations of compound (Table 3). ASDs were stored as per the manufacturer’s recommendations. Prior to commencement of treatments, all ASDs were brought to room temperature, and temperature + humidity was recorded using a Testo 605 IAQ Probe.

**Table 3.** Limit of reporting

Material	Starting quantity	Recovery	LOR
Cefalexin	29 μg/mL	100%	0.02 μg/disc
Cefaclor	34 μg/mL	100%	0.02 μg/disc
Cefuroxime	33 μg/disc	100%	0.02 μg/disc
Cefixime	5.1 μg/disc	100%	0.02 μg/disc

LOR, limit of reporting.

The following treatments were performed as part of the validation phase.

##### Aqueous phase

In the aqueous phase of the study, ASDs preloaded with cephalosporin compounds (*n* = 4 for each of Cefaclor, Cefuroxime, Cephalexin, Cefixime) were exposed to 400 ppm of ClO_2_ (ClorDiSys Solutions, Inc.) in an aqueous solution for 24 h, achieving a cumulative exposure of 9600 ppm-hours. The discs were placed evenly spaced in a Petri dish and immersed in 5 mL of ClO_2_ solution, and following the exposure, samples were neutralized using sodium thiosulfate and subjected to UPLC-MS/MS analysis. No ClO_2_ neutralization steps were carried out on samples before extraction and analysis. Samples were analyzed as received in line with standard analytical practice. Petri dishes were sealed to mitigate loss of ClO_2_ due to off-gassing. This phase was conducted in controlled laboratory conditions at standard temperature and pressure to validate (ClO_2_) efficacy in degrading each compound at 9600 ppm-hours accurately. In the aqueous phase, exact ClO_2 _ concentration over the 24 h could not be measured due to the practical difficulties of measuring the aqueous solution in small volumes; therefore, achievement of 9600 ppm-hours was calculated based on dose and exposure time.

##### Gaseous phase

The gaseous phase involved exposing cephalosporin-impregnated ASDs (Table 3) to dClO_2_ gas (ClorDiSys Solutions, Inc.). A 1.1 m³ fully sealed polymer-based chamber was used, and the concentration of dClO_2_ was maintained at 5mg/L for 9600 ppm-hours, monitored continuously using an in-line gas analyzer (ClorDiSys Solutions, Inc.) and ensured no noticeable drop in gas concentration levels from adsorption or partitioning within the chamber. ASDs (*n* = 5 for each of Cefaclor, Cefuroxime, Cephalexin, Cefixime) were placed at various positions within the chamber to ensure uniform exposure, including difficult-to-reach areas to simulate real-world facility environments. Relative humidity (RH) was raised and kept at 75% to optimize dClO_2_ gas efficacy (Lorcheim, Lorcheim & Czarneski, 2009).^6^ Prior to gassing, ASDs were preconditioned for 45 min at 75% RH. The chemical degradation and biological inactivation of cephalosporin residues were confirmed using UPLC-MS/MS and microbial challenge testing respectively. Post-treatment, no ClO_2_ neutralization steps were carried out on samples before extraction and laboratory analysis. Samples were analyzed as received in line with standard analytical practice.

#### Microbial challenge testing

Post-treatment, a microbial challenge test was performed to determine biological inactivation. For each phase, a number of ASDs for each cephalosporin compound were used, plus untreated controls (aqueous phase—*n* = 4, gassing phase—*n* = 5 for each compound). The treated ASDs and untreated controls, for each compound in all phases, were placed on 90 mm Petri dishes with premade agar media (Southern Biological) and inoculated with live *Escherichia coli* (K-12 strain) using a cell spreader. Each Petri dish was labeled, and all discs were incubated in a laboratory incubator at 37°C for 48 h. Confluent growth was observed on all plates, meeting Clinical and Laboratory Standards Institute criteria for valid zone interpretation.^15^ Growth was observed and recorded by an ISO 17025-accredited laboratory (AMAL Analytical Pty, Australia), measured using a metric ruler under standard practices and photographed.^15^ Photographic images were taken using a handheld camera and are provided in Supplementary Material (Supplementary Data S3 and S4). Images were not used to interpret results.

#### Validation criteria

Decontamination efficacy of chemical degredation was validated through analysis of samples post-treatment (gaseous and aqueous) utilizing the UPLC-MS/MS method developed. Data and statistics supporting the validation methodology, including reproducibility, sensitivities, and detection limits including instrument detection limit, method detection limit, level of quantitation, and limit of reporting (LOR), were provided (Supplementary Data S8).

The degradation to below the LOR was established as the key metric, with successful degradation defined as <0.02 μg/disc (LOR) for all compounds. Biological inactivation, via Microbial Challenge testing, was validated by the presence of bacterial growth within 1 mm of treated ASDs. Presence of bacterial growth was defined as at least one colony present within 1 mm of the disc. Growth or inhibition was assessed using the unaided eye by an independent ISO 17025-accredited microbiology laboratory (AMAL Analytical Pty, Australia), in accordance with internationally accepted antimicrobial susceptibility testing practices.^15^ The minimum distance between the disc edge and first evidence of microbial growth was recorded, with growth at or within 1 mm defined as the key metric for biological inactivation for the purposes of this study.

These dual assessments provided comprehensive validation of both chemical degradation and biological inactivation.

### Verification Phase

#### Facility setup and preparation

The real-world verification phase was conducted in a pharmaceutical manufacturing facility dedicated to the production, packing, and storing of non-sterile oral cephalosporins formulated as capsules, tablets, and powders for oral solutions. The manufacturer terminated the manufacturing of its cephalosporin product portfolio (Cefalexin, Cefaclor, Cefixime, Cefuroxime) for business reasons. This decision created the need to lift the dedicated status of the facility and its equipment and to repurpose them for future non-cephalosporin product manufacturing. The facility is a ground floor building of 450 m^2^ consisting of 23 rooms, of which 16 rooms are operated in good manufacturing practice (GMP) controlled conditions (production, packing, and storage). The remaining rooms consisted of a lobby, changing rooms, sanitary conveniences, and a break room for personnel. The main production and packing equipment used includes a dispensing booth, a colloid mill, a roller compactor, a blender, a capsule machine, a tableting machine, a film coating machine, a primary and secondary packaging machine, a dry syrup powder filling machine, and a serialization and tamper-evident labeling machine. The facility is served by a dedicated Heating, Ventilation, and Air Conditioning (HVAC) system supplying it with 100% fresh air. The HVAC incorporates adequate filtration systems that include HEPA filters in both supply and exhaust streams.

From a regulatory standpoint, the manufacturer held a GMP certificate of compliance and a manufacturing license for the facility. These permits were issued by the National Competent Authority of an European Union member state. Prior to decontamination, the facility was thoroughly mapped to identify areas of potential contamination, particularly hard-to-reach zones such as air ducts, vents, false ceilings, and storage areas. A total of 48 Cefaclor ASDs were strategically placed throughout the facility in both high- and low-risk areas, including both exposed surfaces and those shielded from direct airflow (see Supplementary Data S1 for more detail on the facility). Discs were placed at mid-height across all rooms being decontaminated. ASDs were placed on flat horizontal surfaces including benches, ledges, and equipment housings where accessible; no internal duct placement was performed due to access limitations.

#### ClO_2_ gas application and monitoring

The dClO_2_ decontamination was carried out using a dClO_2_ gas generation system (ClorDiSys Solutions, Inc.), designed to introduce dClO_2_ gas at a concentration of 5 mg/L to achieve 9600 ppm-hours exposure, as previously validated in controlled conditions. The system utilized spectrophotometry, using the ClorDiSys EMS system (ClorDiSys Solutions, Inc.), to continuously monitor both the gas concentration and RH levels within the facility, ensuring that the desired decontamination conditions were maintained throughout the treatment cycle.

#### Verification

Across 24 locations, 24 pairs of Cefaclor ASDs (a total of 48) were placed throughout the facility, which served as dual-function indicators, verifying both chemical degradation and biological inactivation. Cefaclor was selected based on being the only cephalosporin ASD with previous evidence of ClO_2_ inactivation.^5^ Following the completion of the decontamination cycle, the pairs were collected, with one disc from each pair sent for analysis using UPLC-MS/MS for chemical degradation and the other for microbial challenge tests for biological inactivation as per the validation methodology. The successful decontamination of a location was based on meeting the acceptance criteria of both the UPLC-MS/MS analysis and the microbial challenge testing, being labeled a “PASS.”

#### UPLC-MS/MS analysis

In the verification, of the 48 discs in total, 24 discs were sent for chemical analysis to measure the remaining concentrations of cephalosporins. Degradation below the 1 μg/disc was considered the acceptance criteria for a successful outcome during the verification. The LOR was not used as a metric for success for the verification, as the recovery of controls highlighted the presence of 35 μg/disc for the Cefaclor discs placed for the verification study, 1 μg/disc more than the 34 μg/disc defined in the validation phase, meaning the developed test method returned recoveries of over 100%. A 1 μg leniency was defined for the real-world verification to accommodate the larger starting quantity of the Cefaclor discs.

#### Microbial challenge testing

The remaining 24 discs were subjected to microbial challenge testing to confirm biological inactivation. The treated ASDs were placed on 90 mm Petri dishes with premade agar media (Southern Biological) and inoculated with live *E. Coli* (K-12 strain) using a cell spreader. Each Petri dish was labeled, and all discs were incubated in a laboratory incubator at 37°C for 48 h. Growth was observed, measured, and photographed. The presence of bacterial growth at or within 1 mm of the treated discs was confirmed as the acceptance criteria for successful biological inactivation. The presence of bacterial growth is defined as at least one colony present at or within 1 mm of the disc, confirmed visually by an ISO 17025 laboratory (AMAL Analytical Pty, Australia). Please see the section “Validation Criteria” for more details.

#### Method cost analysis

The per-sample cost, turnaround time, and complexity of the LC-MS/MS and microbial challenge testing were provided by AMAL Analytical (Australia) Pty Ltd (Victoria, Australia) and Biosafety (Victoria, Australia) directly. Both providers are ISO 17025 approved for laboratory analytical testing.

#### Statistical methods

To assess the level of agreement between chemical and biological verification methods, we compared results from LC-MS/MS quantification with microbial challenge outcomes for each cephalosporin compound during each phase for validation. For each ASD, the LC-MS/MS result was classified as either “<0.02 μg/disc” (below the LOR) or “≥0.02 μg/disc,” while microbial inactivation was defined as visible *E. coli* growth occurring within 1 mm of the disc edge as “yes” or “no.” Statistical agreement between the two binary outcome sets was assessed using Cohen’s kappa (κ) coefficient, calculated in R (version 4.3.0) using the irr package. This metric accounts for agreement occurring by chance, with κ values ≥0.81 interpreted as “almost perfect agreement.” This approach was selected to reflect the application of microbial challenge testing as a qualitative verification tool and to evaluate its equivalence with quantitative LC-MS/MS under conditions of full degradation.

## Results

An LOR of 0.02 μg/disc was produced for each compound following UPLC-MS/MS method development (Table 3) with 100% recovery. Starting quantities varied between each compound tested, with Cefaclor discs having the heaviest load and Cefixime the least, with each amount differing to the certification (Tables 1 and 3). The results from UPLC-MS/MS and microbial challenge tests during validation showed complete degradation and significant inactivation of cephalosporin residues following exposure to 400 ppm of aqueous ClO_2_ solution for 24 h, equating to 9600 ppm-hours (Table 4 and Supplementary Data S3 and S6). UPLC-MS/MS and microbial challenge tests also showed complete degradation and significant inactivation of each cephalosporin compound when discs were exposed to 9600 ppm-hours of gaseous ClO_2_ (Table 5 and Supplementary Data S4 and S6).

**Table 4.** Summary of aqueous validation results

Material	Replicate	Starting quantities	Limit of reporting (μg/disc)	Amount remaining (μg)	Growth within 1 mm	Pass (yes/no)
Cefalexin	1	29 μg/mL	0.02	<0.02	Yes	Yes
	2	29 μg/mL	0.02	<0.02	Yes	Yes
	3	29 μg/mL	0.02	<0.02	Yes	Yes
	4	29 μg/mL	0.02	<0.02	Yes	Yes
Cefaclor	1	33 μg/mL	0.02	<0.02	Yes	Yes
	2	33 μg/mL	0.02	<0.02	Yes	Yes
	3	33 μg/mL	0.02	<0.02	Yes	Yes
	4	33 μg/mL	0.02	<0.02	Yes	Yes
Cefuroxime	1	33 μg/disc	0.02	<0.02	Yes	Yes
	2	33 μg/disc	0.02	<0.02	Yes	Yes
	3	33 μg/disc	0.02	<0.02	Yes	Yes
	4	33 μg/disc	0.02	<0.02	Yes	Yes
Cefixime	1	5.1 μg/disc	0.02	<0.02	Yes	Yes
	2	5.1 μg/disc	0.02	<0.02	Yes	Yes
	3	5.1 μg/disc	0.02	<0.02	Yes	Yes
	4	5.1 μg/disc	0.02	<0.02	Yes	Yes
Blank	1	0 μg/mL	0.02	<0.02	N/A	
	2	0 μg/mL	0.02	<0.02		

N/A, not available.

**Table 5.** Dry chlorine dioxide gaseous validation summary

Material	Replicate	Starting quantities	Limit of reporting (μg/disc)	Amount remaining (μg)	Growth within 1 mm	Pass (yes/no)
Cefalexin	1	31 μg/disc	0.02	<0.02	Yes	Yes
	2	31 μg/disc	0.02	<0.02	Yes	Yes
	3	31 μg/disc	0.02	<0.02	Yes	Yes
	4	31 μg/disc	0.02	<0.02	Yes	Yes
	5	31 μg/disc	0.02	<0.02	Yes	Yes
Cefaclor	1	34 μg/disc	0.02	<0.02	Yes	Yes
	2	34 μg/disc	0.02	<0.02	Yes	Yes
	3	34 μg/disc	0.02	<0.02	Yes	Yes
	4	34 μg/disc	0.02	<0.02	Yes	Yes
	5	34 μg/disc	0.02	<0.02	Yes	Yes
Cefuroxime	1	33 μg/disc	0.02	<0.02	Yes	Yes
	2	33 μg/disc	0.02	<0.02	Yes	Yes
	3	33 μg/disc	0.02	<0.02	Yes	Yes
	4	33 μg/disc	0.02	<0.02	Yes	Yes
	5	33 μg/disc	0.02	<0.02	Yes	Yes
Cefixime	1	5.1 μg/disc	0.02	<0.02	Yes	Yes
	2	5.1 μg/disc	0.02	<0.02	Yes	Yes
	3	5.1 μg/disc	0.02	<0.02	Yes	Yes
	4	5.1 μg/disc	0.02	<0.02	Yes	Yes
	5	5.1 μg/disc	0.02	<0.02	Yes	Yes

As seen in Table 6, across both aqueous and gaseous validation phases, there was complete agreement between chemical quantification by LC-MS/MS and biological inactivation assessed via microbial challenge testing. All 36 replicates tested returned concordant “PASS” results across both methods. Cohen’s κ coefficient was 1.00 in both phases, indicating perfect inter-method agreement (κ = 1.00, *n* = 16 aqueous; κ = 1.00, *n* = 20 gaseous). No discordant outcomes or partial inhibition zones were observed, reinforcing the equivalence of the microbial assay with standard analytical verification.

**Table 6.** Statistical agreement between liquid chromatography–tandem mass spectrometry and microbial challenge testing

Validation phase	Total replicates	Concordant results	Observed agreement	Cohen’s kappa (κ)	Interpretation
Aqueous	16	16/16	100%	1.00	Perfect agreement
Gaseous	20	20/20	100%	1.00	Perfect agreement

Method cost analysis demonstrated that the microbial challenge method had a turnaround time of 2 days, required low-level expertise, cost ∼£5, required basic instrumentation and provided qualitative results. UPLC-MS/MS has a longer turnaround, required higher level of expertise, had a higher cost per sample by £95 and required significantly more complex instrumentation. However, only UPLC-MS/MS could provide a quantitative result (see Supplementary Data S7).

### Real-World Verification Results

As seen in Table 7, all 24 disc pairs were successfully degraded and inactivated across all areas to within the acceptance criteria of the building, respectively (see Supplementary Data S2). Spectrophotometry results indicate the achievement of 9600 ppm-hours throughout the facility during the cycle (see Supplementary Data S5). UPLC-MS/MS highlighted the presence of a variety of residual concentrations, although all within the 1 μg acceptable limit to achieve a successful decontamination (PASS). Microbial challenge testing showed all discs to have growth at or within 1 mm, confirming inactivation across all discs. Despite all verification samples meeting the acceptance criteria, three samples, 4.12, 4.16, and 4.22, showed some visual antibiotic activity with growth not fully inhibited to the same extent as the other samples.

**Table 7.** Verification results, including ultra-performance liquid chromatography–tandem mass spectrometry and microbial challenge results, with pass criteria based on **<**1 μg/disc and growth within 1 mm

Disc pair	Location	Remaining amount (μg)	Growth	Pass (yes/no)
1	4.1	<0.02	<1 mm	Yes
2	4.2	0.1	<1 mm	Yes
3	4.3	<0.02	<1 mm	Yes
4	4.4	0.03	<1 mm	Yes
5	4.5	0.38	<1 mm	Yes
6	4.6	0.07	<1 mm	Yes
7	4.7	0.05	<1 mm	Yes
8	4.8	0.03	<1 mm	Yes
9	4.9	0.09	<1 mm	Yes
10	4.10	0.04	<1 mm	Yes
11	4.11	0.05	<1 mm	Yes
12	4.12	0.08	<1 mm	Yes
13	4.13	<0.02	<1 mm	Yes
14	4.14	0.11	<1 mm	Yes
15	4.15	0.31	<1 mm	Yes
16	4.16	0.86	<1 mm	Yes
17	4.17	<0.02	<1 mm	Yes
18	4.18	<0.02	<1 mm	Yes
19	4.19	0.19	<1 mm	Yes
20	4.20	<0.02	<1 mm	Yes
21	4.21	<0.02	<1 mm	Yes
22	4.22	0.61	<1 mm	Yes
23	4.23	<0.02	<1 mm	Yes
24	4.24	<0.02	<1 mm	Yes

See Supplementary Material for location details.

## Discussion

This study presents the first real-world validation of a ClO_2_-based decontamination protocol for cephalosporin residues in pharmaceutical facilities, with dual verification via LC-MS/MS and microbial challenge testing. Independent validation of both aqueous and gaseous ClO_2_ exposures confirmed that each method effectively degraded multiple cephalosporins, including Cefalexin, Cefaclor, Cefixime, and Cefuroxime. Notably, this is the first published study to validate the successful degradation and inactivation of Cefixime and Cefuroxime using ClO_2_, expanding the applicability of this approach beyond previously tested compounds.^5,9,14^

Supporting earlier findings by Tancock-Jones et al.,^5^ Cole et al.,^14^ and Lorcheim et al.,^9^ this study reaffirms that a cumulative exposure of 9600 ppm-hours, delivered via either aqueous or gaseous ClO_2_, is sufficient to degrade cephalosporins below the analytical LOR (<0.02 μg/disc). Importantly, this represents the first demonstration of these results in a real-world pharmaceutical manufacturing environment, confirming that the previously established laboratory efficacy of ClO_2_ translates effectively to full-scale operational settings.^5^ These findings substantiate the scalability and practical applicability of gaseous dClO_2_, elevating it from a promising laboratory-based approach to a validated, field-ready decontamination intervention.

The incorporation of microbial challenge testing, alongside LC-MS/MS, strengthens the validation framework by addressing a known limitation of chemical-only verification methods. As beta-lactam residues may retain immunological relevance even below the LOR, particularly in hypersensitive individuals,^3^ the microbial challenge test provides direct confirmation of biological inactivation. Discs treated with ClO_2_ showed significantly reduced bacterial inhibition, indicating loss of pharmacological activity and supporting the safety of repurposed facilities for non-beta-lactam production.

This study is also the first to demonstrate perfect statistical agreement (Cohen’s κ = 1.00) between chemical (LC-MS/MS) and biological (microbial challenge) outcomes across both aqueous and gaseous ClO_2_ treatment phases, augmenting the conclusions of Tancock-Jones et al. in 2024.^10^ This level of concordance affirms that either method can be reliably used to assess decontamination efficacy. Importantly, this finding suggests that microbial challenge testing may serve as a cost-effective, accessible alternative in settings where UPLC-MS/MS instrumentation is unavailable.

A comparison of the two validation techniques, summarized by two ISO 17025-qualified analytical laboratories (Table 7), highlights important practical considerations. LC-MS/MS offers quantitative results but requires higher expertise, complex instrumentation, and longer turnaround times (∼7 days), at a cost of approximately £100 per sample. In contrast, the microbial challenge test offers qualitative outcomes, is easier to execute with basic microbiological equipment, and can be completed in ∼2 days at a cost of £5 per sample. This stark contrast in resources and accessibility suggests that microbial challenge testing is well suited for use in routine monitoring or resource-constrained environments, though not a replacement for quantitative analysis when precision is required.

The implications of these findings are significant. Regulatory bodies such as the US Food and Drug Administration (FDA),^7^ European Medicines Agency (EMA),^11^ and World Health Organisation (WHO)^2^ mandate rigorous cross-contamination controls for beta-lactam manufacturing facilities. The ability to demonstrate both chemical degradation and biological inactivation of residues fulfills not only current expectations but also preemptively addresses potential future regulatory developments. As analytical technologies advance and detection limits become more stringent, manufacturers applying this protocol can demonstrate that they used a risk-based, patient safety-aligned approach that remains valid even as detection thresholds evolve.

This protocol reinforces the concept of establishing a biological endpoint in cleaning validation, a key consideration for sensitive patient populations, and a growing trend in international regulatory discourse. The use of ASDs as a dual-function tool for chemical and biological assessment introduces a streamlined, scalable workflow for facility-wide validation, including hard-to-reach areas typically underserved by traditional swabbing methods.

Despite these strengths, certain limitations must be acknowledged. The study relied on consistent ClO_2_ distribution using gas analyzers and environmental monitors to maintain dosage across a relatively uniform facility layout. In more complex or compartmentalized facilities, variation in airflow dynamics and surface characteristics may necessitate additional validation efforts or modifications to ensure uniform exposure. Furthermore, variability in the starting concentrations of cephalosporin contamination on ASDs highlights a potential limitation in reproducibility.

A key limitation of the microbial challenge methodology is its reliance on visual inspection by an ISO 17025-accredited laboratory. While this approach follows accepted antimicrobial susceptibility testing practice,^15^ zone interpretation is inherently subjective and may vary between analysts, producing error and disagreement. As noted in the section “Methods,” photographic images were provided for illustrative purposes only and were not used to interpret or measure results in alignment with guidelines.^15^

In this study, some discs—notably 4.16 and 4.20—met the <1 mm acceptance criterion, according to ISO 17025 protocols, but showed evidence of residual biological activity in the photographs compared with those with complete inactivation (Supplementary Data), although activity was significantly less than controls. The incorporation of automated zone-reading systems in future work could provide objective, quantitative confirmation, particularly in borderline cases. Augmenting on this study, if photographs are to be included in the future, the incorporation of a visible measurement scale to assist readers in interpreting agar plate images is advised.

The challenge discs used here contained the highest available loading for Cefaclor (∼35 μg/disc), which likely exceeds environmental contamination levels in cleanroom-grade facilities. While this presented a stringent test of dClO_2_ penetration and inactivation capability, it may not reflect typical field conditions. Future studies could first assess in situ contamination levels and then select challenge disc loads representative of the environment, improving the realism of verification trials.

Future studies should aim to define the maximum initial contamination load that this protocol can effectively inactivate. Applying this method to facilities manufacturing different compounds will be limited to the commercial availability of the target ASDs.

The acceptance criterion used here (<1 mm) was intentionally conservative. When microbial challenge testing is used solely as an indicator alongside quantitative LC-MS/MS confirmation, a less stringent threshold (<3 mm) might still provide meaningful assurance while accommodating biological and methodological variability.

Another important direction for future research lies in extending this validation approach to other beta-lactam classes, such as carbapenems. While ClO_2_ has broad oxidative capabilities,^8–10^ degradation pathways and by-products may differ between compounds, and their biological safety should be independently verified. Given the well-documented challenges of cross-reactivity and immune sensitization among beta-lactam-allergic individuals,^1–4,6^ degradation does not necessarily equate to risk elimination unless confirmed by biological testing.

In addition, application of this protocol to facilities with higher contamination risk, such as those producing sterile APIs or highly potent beta-lactams, should be investigated. Integration with adjunctive strategies such as HEPA-filtered air systems, high-shear fogging, or repeat decontamination cycles may be required to address such high-risk settings. No corrosion or other adverse effects on facility infrastructure were observed following dClO_2_ exposure. However, there were cases where non-stainless-steel equipment parts were oxidized. These refer to several chromium-plated tableting punches stored in Room 4.11. Oxidized punches were seen in 5 out of 14 toolboxes located in Room 4.11. Other equipment affected includes the worm gear component of the tableting machine’s turret, a non-stainless-steel, non-product contact part. This tableting machine was stored in Room 4.13. It should be noted that non-stainless-steel tableting machine equipment and components are also sensitive to water exposure. These were disposed of by the company since they have no significant operational or financial value. In any case, the aforementioned tableting machine had been classified by the company as out-of-date equipment, prior to dClO_2_ decontamination, and thus no plans were made for its future applications (see Supplementary Data S9 for more detail). Nevertheless, future work should give consideration to material compatibility and potential ClO_2_ degradation of sensitive equipment over prolonged use, as ClO_2_ has been reported previously to be corrosive to some materials under certain conditions.^16^

## Conclusion

This study establishes a validated, scalable, and operationally feasible protocol for the decontamination of cephalosporin residues in pharmaceutical manufacturing settings to enable repurposing for non-beta-lactam applications. Independent assessment of both aqueous and gaseous ClO_2_ applications demonstrated effective degradation and biological inactivation of all cephalosporins, including Cefixime and Cefuroxime for the first time. The protocol’s dual verification approach, combining quantitative LC-MS/MS analysis with qualitative microbial challenge testing, confirms its robustness and adaptability across varying resource environments. The observed concordance (Cohen’s κ = 1.00) between these methodologies reinforces their complementary utility in contamination control strategies. In alignment with current regulatory expectations and anticipating future analytical sensitivities, this protocol provides a scientifically defensible and patient safety-oriented framework for the repurposing of beta-lactam manufacturing facilities. Its implementation supports enhanced biosafety assurance and operational flexibility in compliance-driven pharmaceutical production environments.

## Authors’ Contributions

R.T.-J.: Conceptualization, writing—original draft, writing—review and editing, and data curation. S.K.: Conceptualization, funding acquisition, supervision, writing—review and editing, project administration, and resources. B.C. and J.M.: Formal analysis, methodology, and writing—review and editing.

## Supplemental Material

sj-docx-1-apb-10.1177_15356760251388996 — Supplemental material for Repurposing Success: Real-World Validation of a Novel Cephalosporin Decontamination Protocol Using Chlorine Dioxide and Disc Diffusion Testing Supplemental material, sj-docx-1-apb-10.1177_15356760251388996 for Repurposing Success: Real-World Validation of a Novel Cephalosporin Decontamination Protocol Using Chlorine Dioxide and Disc Diffusion Testing by

sj-docx-1-apb-10.1177_15356760251388996 — Supplemental material for Repurposing Success: Real-World Validation of a Novel Cephalosporin Decontamination Protocol Using Chlorine Dioxide and Disc Diffusion Testing Supplemental material, sj-docx-1-apb-10.1177_15356760251388996 for Repurposing Success: Real-World Validation of a Novel Cephalosporin Decontamination Protocol Using Chlorine Dioxide and Disc Diffusion Testing by

sj-docx-1-apb-10.1177_15356760251388996 — Supplemental material for Repurposing Success: Real-World Validation of a Novel Cephalosporin Decontamination Protocol Using Chlorine Dioxide and Disc Diffusion Testing Supplemental material, sj-docx-1-apb-10.1177_15356760251388996 for Repurposing Success: Real-World Validation of a Novel Cephalosporin Decontamination Protocol Using Chlorine Dioxide and Disc Diffusion Testing by

sj-docx-1-apb-10.1177_15356760251388996 — Supplemental material for Repurposing Success: Real-World Validation of a Novel Cephalosporin Decontamination Protocol Using Chlorine Dioxide and Disc Diffusion Testing Supplemental material, sj-docx-1-apb-10.1177_15356760251388996 for Repurposing Success: Real-World Validation of a Novel Cephalosporin Decontamination Protocol Using Chlorine Dioxide and Disc Diffusion Testing by

sj-docx-1-apb-10.1177_15356760251388996 — Supplemental material for Repurposing Success: Real-World Validation of a Novel Cephalosporin Decontamination Protocol Using Chlorine Dioxide and Disc Diffusion Testing Supplemental material, sj-docx-1-apb-10.1177_15356760251388996 for Repurposing Success: Real-World Validation of a Novel Cephalosporin Decontamination Protocol Using Chlorine Dioxide and Disc Diffusion Testing by

sj-docx-1-apb-10.1177_15356760251388996 — Supplemental material for Repurposing Success: Real-World Validation of a Novel Cephalosporin Decontamination Protocol Using Chlorine Dioxide and Disc Diffusion Testing Supplemental material, sj-docx-1-apb-10.1177_15356760251388996 for Repurposing Success: Real-World Validation of a Novel Cephalosporin Decontamination Protocol Using Chlorine Dioxide and Disc Diffusion Testing by

sj-docx-1-apb-10.1177_15356760251388996 — Supplemental material for Repurposing Success: Real-World Validation of a Novel Cephalosporin Decontamination Protocol Using Chlorine Dioxide and Disc Diffusion Testing Supplemental material, sj-docx-1-apb-10.1177_15356760251388996 for Repurposing Success: Real-World Validation of a Novel Cephalosporin Decontamination Protocol Using Chlorine Dioxide and Disc Diffusion Testing by

sj-docx-1-apb-10.1177_15356760251388996 — Supplemental material for Repurposing Success: Real-World Validation of a Novel Cephalosporin Decontamination Protocol Using Chlorine Dioxide and Disc Diffusion Testing Supplemental material, sj-docx-1-apb-10.1177_15356760251388996 for Repurposing Success: Real-World Validation of a Novel Cephalosporin Decontamination Protocol Using Chlorine Dioxide and Disc Diffusion Testing by

sj-docx-1-apb-10.1177_15356760251388996 — Supplemental material for Repurposing Success: Real-World Validation of a Novel Cephalosporin Decontamination Protocol Using Chlorine Dioxide and Disc Diffusion Testing Supplemental material, sj-docx-1-apb-10.1177_15356760251388996 for Repurposing Success: Real-World Validation of a Novel Cephalosporin Decontamination Protocol Using Chlorine Dioxide and Disc Diffusion Testing by
