# Supplementary material for: Repurposing Success: Real-World Validation of a Novel Cephalosporin Decontamination Protocol Using Chlorine Dioxide and Disc Diffusion Testing
Source: Appl Biosaf. 2025 Nov 13;31(3):214–23. doi: 10.1177/15356760251388996 (PMC13443250; doi:10.1177/15356760251388996)
Supplement: Supplementary file 1 [file sj-docx-1-apb-10.1177_15356760251388996.docx]

Supplementary Material: Facility Layout and disc-pair locations (numbers from 4.1-4.23). To synchronise with the table 8 and supplementary material 2 data, the image below include an area named 4.9A, which in table 8 and SM2 equates to 4.10. All subsequent locations to 4.9A seen below refer to the sample in table 8 and SM2 which is one above it`s location on the figure below. Exact location vs sample number: location 4.10= sample 4.11, location 4.11= sample 4.12, location 4.12=sample 4.13, location 4.13=sample 4.14, location 4.14=sample 4.15, location 4.15= sample 4.16, location 4.16=sample 4.17, location 4.17=sample 4.18, location 4.18-sample 4.19, location 4.19=sample 4.20, location 4.20=sample 4.21, location 4.21=sample 4.22, location 4.22=sample 4.23, location 4.23=sample 4.24 .
