# Supplementary material for: Repurposing Success: Real-World Validation of a Novel Cephalosporin Decontamination Protocol Using Chlorine Dioxide and Disc Diffusion Testing
Source: Appl Biosaf. 2025 Nov 13;31(3):214–23. doi: 10.1177/15356760251388996 (PMC13443250; doi:10.1177/15356760251388996)
Supplement: Supplementary file 2 [file sj-docx-2-apb-10.1177_15356760251388996.docx]

Supplementary Material: Validation Method Development Data

|  | **Cefalexin** | **Cefaclor** | **Cefuroxime** | **Cefixime** |
| --- | --- | --- | --- | --- |
| **5ppb - Area count - Run 1** | 5700 | 2628 | 2704 | 2169 |
| **5ppb - Area count - Run 2** | 6025 | 2716 | 2769 | 2129 |
| **5ppb - Area count - Run 3** | 5630 | 2642 | 2548 | 2143 |
| **5ppb - Area count - Run 4** | 5712 | 2719 | 2564 | 2366 |
| **5ppb - Area count - Run 5** | 5844 | 2835 | 2297 | 2474 |
| **5ppb - Area count - Run 6** | 6075 | 2866 | 2348 | 2227 |
| **5ppb - Area count - Run 7** | 6168 | 2999 | 2299 | 2504 |
| **5ppb - Area count - Run 8** | 5993 | 2792 | 2340 | 2467 |
| **5ppb - Area count - Run 9** | 6066 | 2875 | 2307 | 2387 |
| **5ppb - Area count - Run 10** | 6078 | 2879 | 2330 | 2422 |
|  |  |  |  |  |
| **Standard deviation** | 191 | 118 | 180 | 147 |
| **Mean** | 5929 | 2795 | 2451 | 2329 |
|  |  |  |  |  |
|  |  |  |  |  |
| **Calibration Curve R2** | 0.998425 | 0.998354 | 0.99983 | 0.995906 |
| **Intercept** | 1447.59 | -317.159 | -69.8955 | 665.31 |
| **Slope** | 1469170 | 649783 | 529718 | 425325 |
|  |  |  |  |  |
| **Instrument detection limit (IDL)** | 0.000595 | 0.001032 | 0.001153 | 0.000527 |
| **Method detection limit (MDL)*** | 0.0030 | 0.0052 | 0.0058 | 0.0026 |
| **Level of Quantitation (LOQ)** | 0.0089 | 0.0155 | 0.0173 | 0.0079 |
| **Level of Reporting (LOR)** | 0.02 | 0.02 | 0.02 | 0.02 |
|  |  |  |  |  |
| *MDL based on x20 dillution of samples against IDL | | |  |  |
| **LOQ is calcualted as MDL x3 |  |  |  |  |
| ***LOR must be equal to or greater than LOQ | |  |  |  |

| Disc properties | | | | | |
| --- | --- | --- | --- | --- | --- |
| **Compound** | **Type** | **Supplier** | **Lot** | **Expiry** | **Concentration (ug)** |
| Cefixime | Disc | Bio-Rad | 64541034 | 24/04/2025 | 5 |
| Cefalexin | Disc | Bio-Rad | 64539676 | 11/04/2025 | 30 |
| Cefuroxime | Disc | Bio-Rad | 64530356 | 19/02/2025 | 30 |
| Cefaclor | Disc | Oxoid | 3581543 | 17/11/2023 | 30 |

| Spiking summary | | | | | | | | |
| --- | --- | --- | --- | --- | --- | --- | --- | --- |
| **Compound** | **Batch 43180** | **Batch 43180 2** | **Batch 43210** | **Batch 432103** | **Batch 43244** | **Batch 432444** | **Batch 43370** | **Batch 43439** |
| Cefixime | 86% | 80% |  |  |  |  | 102% | 140*% |
| Cefalexin | 100% | 97% |  |  | 103% |  | 103% | 97% |
| Cefuroxime | 97% | 90% |  |  |  |  | 110% | 117% |
| Cefaclor | 107% | 103% | 117% | 103% |  | 91% | 113% | 110% |
| *Outlier |  |  |  |  |  |  |  |  |

| Statistical Summary | | | | |
| --- | --- | --- | --- | --- |
| **Compound** | **Mean Recovery** | **Standard Deviation** | **Number, n** | **Reproducability, as RSD %** |
| Cefixime | 89% | 0.114 | 3 | 13% |
| Cefalexin | 100% | 0.033 | 5 | 3% |
| Cefuroxime | 103% | 0.122 | 4 | 12% |
| Cefaclor | 106% | 0.084 | 7 | 8% |
