# Supplementary material for: Repurposing Success: Real-World Validation of a Novel Cephalosporin Decontamination Protocol Using Chlorine Dioxide and Disc Diffusion Testing
Source: Appl Biosaf. 2025 Nov 13;31(3):214–23. doi: 10.1177/15356760251388996 (PMC13443250; doi:10.1177/15356760251388996)
Supplement: Supplementary file 3 [file sj-docx-3-apb-10.1177_15356760251388996.docx]

Supplementary Material: Spectrophotometry graphs from the real-world verification phase. Exposure(above) and Concentration(below) graphs are provided.

ppm-hours

Time (hh;mm;ss)

Time (hh;mm;ss)

Concentration (mg/L)
