# Supplementary material for: Repurposing Success: Real-World Validation of a Novel Cephalosporin Decontamination Protocol Using Chlorine Dioxide and Disc Diffusion Testing
Source: Appl Biosaf. 2025 Nov 13;31(3):214–23. doi: 10.1177/15356760251388996 (PMC13443250; doi:10.1177/15356760251388996)
Supplement: Supplementary file 5 [file sj-docx-5-apb-10.1177_15356760251388996.docx]

Supplementary Material: Gaseous phase microbial challenge results post-treatment(left) vs untreated controls (Right).

**Microbial Challenge results Cefuroxime. Control discs(right side) were not exposed to dClO2.**

**Microbial Challenge results Cefixime. Control discs(right side) were not exposed to dClO2.**

**Microbial Challenge results Cefaclor. Control discs(right side) were not exposed to dClO2.**

**Microbial Challenge results Cefalexin. Control discs(right side) were not exposed to dClO2.**
