# Supplementary material for: Repurposing Success: Real-World Validation of a Novel Cephalosporin Decontamination Protocol Using Chlorine Dioxide and Disc Diffusion Testing
Source: Appl Biosaf. 2025 Nov 13;31(3):214–23. doi: 10.1177/15356760251388996 (PMC13443250; doi:10.1177/15356760251388996)
Supplement: Supplementary file 6 [file sj-docx-6-apb-10.1177_15356760251388996.docx]

Supplementary Material: Aqueous phase microbial challenge results post-treatment (above) vs untreated controls (below).

**Cefalexin**

**Cefaclor**

**Cefuroxime**

**Cefixime**

**Cefaclor**

**Cefuroxime**

**e**

**Cefalexin**

**Cefixime**
