# Supplementary material for: Repurposing Success: Real-World Validation of a Novel Cephalosporin Decontamination Protocol Using Chlorine Dioxide and Disc Diffusion Testing
Source: Appl Biosaf. 2025 Nov 13;31(3):214–23. doi: 10.1177/15356760251388996 (PMC13443250; doi:10.1177/15356760251388996)
Supplement: Supplementary file 7 [file sj-docx-7-apb-10.1177_15356760251388996.docx]

| **Method** | **Turn-around time** | **Required Expertise** | **Cost Per Sample** | **Result output** | **Instrumentation Requirements.** |
| --- | --- | --- | --- | --- | --- |
| UPLC-MS/MS | 7 days (including method development and running LC-MS/MS) | High | ~£100 | Quantitative | Complex |
| Microbial Challenge | 2 days ( including plate preparation & culture) | Low | ~£5 | Qualitative | Basic |

**Table. Per-sample Costing, Turn-around times, method complexity and expertise requirements and result output.**
