# Supplementary material for: Repurposing Success: Real-World Validation of a Novel Cephalosporin Decontamination Protocol Using Chlorine Dioxide and Disc Diffusion Testing
Source: Appl Biosaf. 2025 Nov 13;31(3):214–23. doi: 10.1177/15356760251388996 (PMC13443250; doi:10.1177/15356760251388996)
Supplement: Supplementary file 8 [file sj-docx-8-apb-10.1177_15356760251388996.docx]

**Supplementary material:** Chromatographic overlay of all target analytes in all samples processed during study and the lowest level instrumental control sample (concentration = 0.005ppm). The clear response of analytes in the control standard demonstrates the degree of loss experienced in pre-inoculated discs during the procedure.

Four peaks represent the control samples:

1.) Peak 1 – 1.20min - Cefaclor
2.) Peak 2 – 1.35min - Cefalexin
3.) Peak 3 – 1.47min - Cefixime
4.) Peak 4 – 1.75min – Cefuroxime
