# Supplementary material for: Repurposing Success: Real-World Validation of a Novel Cephalosporin Decontamination Protocol Using Chlorine Dioxide and Disc Diffusion Testing
Source: Appl Biosaf. 2025 Nov 13;31(3):214–23. doi: 10.1177/15356760251388996 (PMC13443250; doi:10.1177/15356760251388996)
Supplement: Supplementary file 9 [file sj-docx-9-apb-10.1177_15356760251388996.docx]

Supplementary material: Material Compatibility

The images below provide a visual representation of some of the oxidation witnessed on some of the equipment in rooms 4.11 and 4.13.
